# Acknowledgment of Reviewers

**DOI:** 10.1002/aps3.11319

**Published:** 2020-01-15

The editors are deeply grateful to the following reviewers (members and nonmembers of the Botanical Society of America) who have generously given of their time to review manuscripts submitted to *Applications in Plant Sciences*. The list includes those who reviewed manuscripts from December 31, 2018, to December 31, 2019. If any reviewer's name has been omitted, we apologize, and ask that a note be sent to the Managing Editor so the list can be corrected.

Alba, Christina

Amici, Autumn

Atherton, Jon

Bandyopadhyay, Tirthankar

Baranov, Sergey

Barrett, Craig

Basu, Saikat

Bergstrom, Aaron

Berra, Elias Fernando

Bonnet, Pierre

Botanga, Chris

Cahoon, Edgar

Callihan, Alexia

Carlson, Craig

Chambers, Sally

Chen, Kimberly

Clarke, Jennifer

Coiro, Mario

Colom, Sara

Crimmins, Theresa

Dean, Gillian

Denny, Ellen

de Soto Suárez, Lucía

Domozych, David

Earles, Mason

Eaton, Deren

Ellwood, Elizabeth

Eserman, Lauren

Estep, Matt

Farrar, Kerrie

Filloramo, Gina

Gehan, Malia

Genin, Stéphane

Giri, Jitender

Gladish, Daniel

Gomez, Francisco

González, Antonio

Greenwood, David

Guralnick, Rob

Habib, Ayman

Hamilton, Paul

Harbert, Robert

Hardoim, Pablo

Heberling, Mason

Hepler, Nathan

Heuschele, Deborah Jo

Hodel, Richard

Hosseinalizadeh Nobarinezhad, Mahboubeh

Hou, Siyu

Howard, Aaron

Hufft, Rebecca A.

Inglada, Jordi

Inouye, David

Jagiełło, Radosław

Janes, Jasmine

Johnson, Matthew

Jud, Nathan

Kariyat, Rupesh

Kaur, Dalvir

Kenchanmane Raju, Sunil Kumar

Khaki, Saeed

Kiirika, Leonard

Kuhlgert, Sebastian

Kuo, Li‐Yaung

Kuzmina, Maria

Lagomarsino, Laura

Latvis, Maribeth

Leitão, Carlos

Livingston, Sam

Macklin, James

Mandel, Jennifer

Marck, Isaac

Martine, Christopher

Marx, Hannah

Mason, Chase

Maughan, Jeff

Maya‐Lastra, Carlos

Mazer, Susan

McGlaughlin, Mitchell

McKain, Michael

Meerdink, Susan

Metscher, Brian

Metzgar, Jordan

Miller, Nathan

Morales‐Briones, Diego

Mozzherin, Dmitry

Murrell, Zack

Musselman, Lytton

Nelson, Gil

Ollerton, Jeff

Pace, Matthew

Pandey, Bipin Kumar

Park, Daniel

Park, Isaac

Pasiche‐Lisboa, Carlos

Pec, Gregory

Picó, Xavier

Pinson, Jerald

Ploughe, Laura

Pokorny, Lisa

Polo, Jose

Primack, Richard

Priya, Piyush

Ramaraj, Thiru

Ray, Dustin

Ruck, Elizabeth

Rukam, Tomar

Scarcelli, Nora

Schori, Melanie

Scott, Alison

Seago, James

Singh, Daljit

Smith, Mark

Soltis, Douglas

Stavness, Ian

Steinbrenner, Adam

Straub, Shannon

Stubbs, Rebecca

Sweeney, Patrick

Symonds, Vaughan

Telea, Alexandru

Thadeo, Marcela

Thessen, Anne

Thiers, Barbara

Topp, Christopher

Tunison, Robert

VanBuren, Robert

Vargas, Oscar M.

Vemanna, Ramu S.

Wadgymar, Susana

Walker, Joseph

Weitemier, Kevin

White, Alexander

Whitton, Jeannette

Winkler, Daniel

Xu, Yuhang

Yeung, Edward

Zhao, Yanxin

